# Scavenger Receptors as Biomarkers and Therapeutic Targets in Cardiovascular Disease

**DOI:** 10.3390/cells9112453

**Published:** 2020-11-10

**Authors:** Gary A. Cuthbert, Faheem Shaik, Michael A. Harrison, Sreenivasan Ponnambalam, Shervanthi Homer-Vanniasinkam

**Affiliations:** 1 Faculty of Medicine and Health, University of Leeds, Leeds LS2 9JT, UK; s.homer-v@ucl.ac.uk; 2 School of Molecular & Cellular Biology, University of Leeds, Leeds LS2 9JT, UK; f.shaik@qmul.ac.uk (F.S.); s.ponnambalam@leeds.ac.uk (S.P.); 3 School of Biomedical Sciences, University of Leeds, Leeds LS2 9JT, UK; m.a.harrison@leeds.ac.uk

**Keywords:** scavenger receptors, atherosclerosis, cardiovascular disease, lipids

## Abstract

The process of atherosclerosis leads to the formation of plaques in the arterial wall, resulting in a decreased blood supply to tissues and organs and its sequelae: morbidity and mortality. A class of membrane-bound proteins termed scavenger receptors (SRs) are closely linked to the initiation and progression of atherosclerosis. Increasing interest in understanding SR structure and function has led to the idea that these proteins could provide new routes for cardiovascular disease diagnosis, management, and treatment. In this review, we consider the main classes of SRs that are implicated in arterial disease. We consider how our understanding of SR-mediated recognition of diverse ligands, including modified lipid particles, lipids, and carbohydrates, has enabled us to better target SR-linked functionality in disease. We also link clinical studies on vascular disease to our current understanding of SR biology and highlight potential areas that are relevant to cardiovascular disease management and therapy.

## 1. Introduction

### 1.1. Oxidized Low-Density Lipoprotein and Atherogenesis

**Figure 1:**
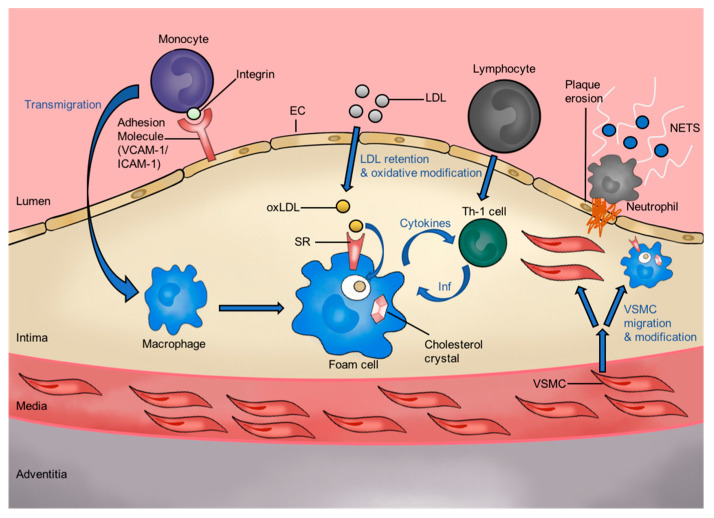
Key events in atherosclerotic plaque initiation. Blood LDL can accumulate within the arterial wall, in the sub-endothelial intima. This accumulated LDL can be chemically modified or oxidized: this new lipid particle species (e.g., oxLDL) promotes chronic inflammation, which promotes the trans-endothelial migration of immune cell types and foam cell development. Abbreviations: endothelial cell, EC; intercellular adhesion molecule-1, ICAM-1; interferon-γ, IFNγ; low-density lipoprotein, LDL; Neutrophil extracellular traps, NETS; oxidized low-density lipoprotein, oxLDL; scavenger receptor, SR; T- helper 1 cell, Th1; vascular cell adhesion molecule-1, VCAM-1; vascular smooth muscle cell, VSMC.

Atherosclerosis is the main biological process underlying cardiovascular disease (CVD) development and pathology [1]. Atherosclerosis is a multifactorial, progressive, and chronic inflammatory process characterized by the accumulation of lipid and fibrous elements within the arterial wall, manifesting macroscopically as an atherosclerotic plaque (Figure 1). The key events in atherosclerotic plaque initiation and progression are depicted in Figure 1. Atherosclerosis is initiated by abnormally high circulating blood low-density lipoprotein (LDL) levels (LDL-cholesterol >40 mg/dL) [2], leading to LDL accumulation within the sub-endothelial matrix of arterial blood vessel walls, usually operating at a higher blood pressure of 80–120 mm Hg [3]. The LDL deposits within arterial vessel walls become chemically modified by free radicals produced by vascular cells, such as the endothelium, vascular smooth muscle, and infiltrating immune cells. These new lipid particle species are termed minimally modified LDL (mmLDL), modified LDL (mLDL), and oxidized LDL (oxLDL). Notably, oxLDL promotes a powerful proinflammatory response by different cell types, including the production of cell surface adhesion molecules and chemotactic factors by the endothelium. OxLDL indirectly promotes the recruitment of circulating blood monocytes by binding to the activated endothelium followed by trans-endothelial migration (TEM) into the sub-endothelial intima, where the monocytes differentiate into macrophages. Here, the accumulation of oxLDL within the vascular wall further causes macrophage scavenger receptors (SRs) to bind and promote oxLDL internalization, leading to the development of lipid-laden foam cells, i.e., the early stages of atherosclerosis (atherogenesis) [4,5]. The vascular smooth muscle cell (VSMC) layer underlying the intima can also respond to endothelial-derived factors secreted in response to oxLDL accumulation [6]. VSMCs also express SRs, which can enable oxLDL internalization and contribution to more lipid-laden foam cell development, albeit to a lesser extent than macrophages [6].

### 1.2. The Role of Atherosclerotic Plaque in CVD

The clinical consequences of atherosclerosis relate principally to ischaemia and acute thrombo-occlusive events at the sites of plaque deposition. Thrombo-occlusive events in the majority of cases result from plaque rupture [7]. The progression of atherosclerotic plaque from early asymptomatic lesions to those which are high risk of rupture (i.e., vulnerable plaque) is well described [8,9]. Vulnerable plaque is characterized by a large necrotic core with an overlying thin fibrous cap heavily infiltrated by foamy macrophages [10]. Observational studies suggest that expansion of the necrotic core precedes plaque rupture [10,11]. Inter-plaque haemorrhage has been identified as a critical step leading to expansion of the necrotic core, where the accumulation of erythrocytes within the core leads to an increased free cholesterol content and excessive macrophage infiltration [8]. Subsequent plaque rupture exposes leukocytes, platelets, and circulating blood factors to underlying thrombogenic proteins, e.g., Von Willebrand factor (VWF), leading to thrombus formation. Recent studies have highlighted the phenomena of coronary artery plaque erosion (without rupture) and the formation of “white thrombus” in patients with acute coronary syndrome (ACS) [12,13]. Plaque erosion has been previously understood to account for one fifth of ACS; however, the most recent data suggests this has increased to one third [14]. The working hypothesis to explain the rise of plaque erosion in ACS is that advances in lipid control have led to a shift in plaque morphology from the classical lipid-rich rupture-prone plaque to a plaque that is low in lipid content and rich in proteoglycans, glycosaminoglycans, and VSMCs. The mechanism of thrombus formation in eroded plaque differs from ruptured plaque. Briefly, the activation and subsequent desquamation and apoptosis of endothelial cells due to disturbed flow leads to the recruitment of neutrophils to the exposed basement membrane. Recruited neutrophils congregate and in turn degranulate, leading to the formation of neutrophil extracellular traps (NETs). NETs comprise mainly of myeloperoxidase and tissue factor amongst other extruded proteins, all of which contribute to the formation of leucocyte-rich white thrombus by promoting leucocyte and platelet recruitment [12,13,14]. The formation of NETs may also be promoted by circulating oxLDL as shown in a recently published in vitro study [15]. In addition, the authors found that oxLDL and NETs exerted synergistic proinflammatory effects on endothelial cells.

### 1.3. A Brief Introduction to SRs

**Figure 2:**
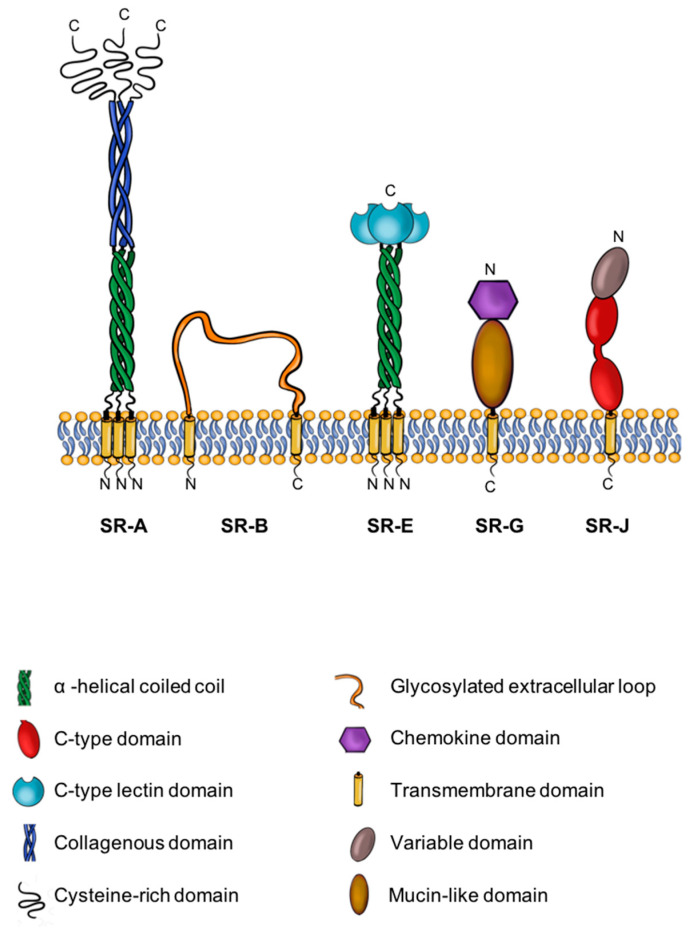
Five different SR classes (A, B, E, G, J) are most closely linked to the initiation and progression of atherosclerosis. These molecules are potential biomarkers and therapeutic targets. Their structures are represented in the sketches above. Abbreviations: N-terminus, N; C-terminus, C.

SRs are defined as integral membrane proteins present at the cell surface, which typically bind multiple ligands to promote clearance or harmful non-self or altered-self substance. SR activity usually requires sensing and/or internalization of such substances, which ultimately leads to the elimination of these substances [16]. In 1979, Goldstein, Brown and co-workers first described scavenger receptors (SRs) whilst investigating how low-density lipoprotein (LDL) particles promote foam cell development in atherosclerosis [17,18]. This led to the discovery that macrophages exhibit high-affinity binding sites, which enable recognition of modified and negatively charged LDL particles, thus promoting lipid particle internalization and subsequent formation of lipid-enriched foam cells [18]. Different SRs have since been identified and categorized into 10 distinct classes based on primary structure (Table 1) [19]. SRs are defined by common properties, such as the recognition and binding to ligands, such as lipoprotein particles, phospholipids, apoptotic cells, carbohydrates, and cholesterol esters; however, the primary consensus protein sequence within each class bears little or no homology to other classes [19]. SRs are expressed largely in vascular and immune cells under physiological or pathological conditions. Different immune cells express SRs, such as macrophages, which are linked to the initiation and progression of atherosclerosis. The wide spectrum of SR expression has been linked to pathological conditions, such as degenerative brain disease [20,21], malignancy [22], and systemic inflammatory states, such as sepsis [23], rheumatoid arthritis [24], and renal vasculitis [25]. SR function in regulating atherosclerosis is a major focus of research in CVD diagnosis and therapy. Current work on SR regulation of atherosclerosis focuses largely on five SR classes: SR-A; SR-B (CD36 (cluster of differentiation 36)); SR-E (lectin-like oxidized LDL receptor-1 (LOX-1)); SR-G (CXCL16); SR-J (receptor for advanced glycation end-products (RAGEs)) (Figure 2). This review article aims to highlight SR properties and functionality in the diagnosis and treatment of atherosclerosis linked to CVD as summarized in Table 1.

## 2. SRs as Biomarkers in CVD

The association of SRs with CVD status is well established, and multiple genes and isoforms have been highlighted as potential markers for use in genetic screening for disease risk, or as diagnostic or prognostic biomarkers. Screening patients with a genetic predisposition to CVD has potentially far-reaching benefits in disease prevention and early management. Polymorphisms in genes encoding both CD36 and LOX-1 proteins have been associated with increased overall CVD risk [58,59,60]. A single-nucleotide polymorphism (SNP) of the *OLR1* (*LOX-1, SR-E1*) allele has been shown to increase steady-state LOX-1 levels in vitro, suggesting that this polymorphism may contribute to disease initiation and/or progression; *OLR1* genotyping could be used for CVD risk stratification [60]. Moreover, a study of a group of 150 patients with acute myocardial infarction (MI) vs. a control group of 103 healthy individuals identified SNPs in *OLR1* associated with increased risk of MI [61]. Cohort studies comparing healthy individuals and coronary artery disease (CAD) patient groups have shown association between *CD36* polymorphisms with increased lipid oxidation, CVD risk, and accelerated development of CAD [58,59,62].

Much has been done to investigate the diagnostic value of quantifying SR levels in ACS. Circulating concentrations of soluble SR-derived proteins in serum, and SR levels in specific immune cell types have been studied. The use of an “SR-A index” quantifies monocyte SR-A expression in peripheral blood smears from patients with CAD: this demonstrates that an increase in this SR-A index is associated with altered atherosclerotic plaque status, leading to destabilization, i.e., disruption, fissure, erosion [63]. Furthermore, such an SR-A index could be used to stratify plaque morphology, helping to inform therapeutic strategies for CAD [64,65]. Similar studies show an association between circulating monocyte CD36 levels and atheroma burden in CAD patients [66]. Overexpressed SR-A in macrophages in atherosclerotic plaque has been targeted using SR-A-specific nanoparticles in an apolipoprotein-E (*APO-E*)-null mouse model in order to identify vulnerable plaques using magnetic resonance imaging with some success [67]. A similar study using CD36-targeted nanovesicles to quantify plaque burden in low-density lipoprotein receptor (*LDLR*)-null mice showed similarly promising results [68]. The use of SR-targeted probes in plaque identification, risk stratification, and quantification is an interesting avenue of ongoing research.

Measuring biomarkers in serum is widely used in clinical settings to ACS diagnosis and stratification for treatment [69]. Proteolytically derived soluble fragments or splice isoforms of CD36, LOX-1, CXCL16, and RAGE are potential biomarkers for a range of CVD conditions, including ACS, cardiac failure, cardiomyopathy, and carotid artery disease. A summary of studies investigating the this potential application is seen in Table 2. Baseline serum levels of soluble CD36 (sCD36) have been shown in three recent cohort studies to have no association with the development of CAD in healthy subjects or plaque burden in known CAD patients [70,71]. Baseline levels of sCD36 were also found to not be associated with carotid atherosclerosis in a recently performed cross-sectional study [72]. In contrast, a recent case control study of patients with CAD found an association between increased sLOX-1 levels and burden of CAD, compared with healthy controls [73]. A similar study of metabolic syndrome patients found that baseline sLOX-1 levels were higher in patients with CAD, implying a potential benefit in the measurement of baseline sLOX-1 levels in at-risk cohorts to risk stratify for CAD development [74].

A small clinical study on 67 patients found that serum-soluble LOX-1 (sLOX-1) levels increased significantly in ACS patients compared to a group of patients with stable angina pectoris [75]. A combined index of serum sLOX-1 and cardiac troponin (cTn) levels improved diagnostic accuracy in ACS compared with diagnostic tests carried out in isolation. The same study also found that measuring sLOX-1 levels in ACS was a reliable test compared to a standard diagnosis of ACS [76]. A study on 107 patients undergoing coronary stenting found that measuring sLOX-1 levels was more informative and accurate compared with cTn measurements in ACS diagnosis; a combined sLOX-1/cTn index was a significantly better biomarker tool than measuring sLOX-1 levels alone [77]. The potential ability of sLOX-1 in identifying the most severe acute coronary events was demonstrated in a recently published study comparing levels of sLOX-1 in aspirated coronary thrombus from non-ST elevation and ST-elevation myocardial infarction patients [78]. The studies that compare cTn and sLOX-1 as biomarkers for ACS have consistently shown that sLOX-1 levels are raised earlier compared with cTn [76,77]. The hypothesis to explain this phenomenon is that sLOX-1 is released at the point of plaque rupture and platelet activation during thrombus formation. Based on this hypothesis and the findings from Lee et al. [78], there may be a role for sLOX-1 as a marker for coronary micro-thrombosis peri-procedurally at the point of percutaneous coronary intervention; however, there are no data currently to support this. Baseline sLOX-1 levels may also be of benefit as a prognostic biomarker in stable CAD. A recently published study of 833 stable CAD patients found a positive correlation between baseline sLOX-1 levels and major adverse cardiovascular events [79]. The relationship between LOX-1 and myocardial reperfusion injury has been investigated in several mouse models of myocardial ischaemia; however, this has not been further investigated in human studies. Mouse models have demonstrated that *LOX-1*-null mice exhibit significantly less reperfusion injury and significantly preserve cardiac function compared with wild-type mice [80,81]. In vitro and in vivo heart failure models suggest sLOX-1 release by cardiac myocytes promotes apoptosis [82,83]. To evaluate sLOX-1 as a biomarker for systolic heart failure, serum sLOX-1 levels were measured in 55 patients with systolic heart failure and compared to a control group of 25 individuals. Serum sLOX-1 levels were found to negatively correlate with left ventricular ejection fraction, particularly in patients with ischemia-related aetiology [84]. The relationship between LOX-1 and C-reactive protein (CRP) in atherogenesis and their role as biomarkers for cardiovascular events is an area of ongoing interest. CRP is a widely used biomarker for inflammation, which is also highly predictive of recurrent cardiovascular events [85]. In vitro studies have demonstrated that CRP induces endothelial cell LOX-1 expression, leading to increased monocyte adhesion and oxLDL uptake [86]. The release of sLOX-1 from monocytes is stimulated by incubation CRP, via the activation of tumour necrosis factor-α in vitro [87]. These studies demonstrate the synergistic effects of CRP and LOX-1 and their potential for use as biomarkers in tandem. The evidence supporting the potential use of sLOX-1 as a biomarker for stroke is mounting but is not as significant compared with CAD and ACS. Multiple small cohort studies comparing serum sLOX-1 levels between patients presenting with acute stroke and controls found that serum sLOX-1 levels were significantly raised in ischaemic stroke and haemorrhagic stroke, and that higher sLOX-1 levels independently predicted poorer neurological outcomes [88,89,90]. A recently published prospective population study of 4703 participants found that higher baseline sLOX-1 levels were associated with an increased incidence of ischaemic stroke over a 16.5-year mean follow-up [91]. In participants requiring carotid endarterectomy (*n* = 202), higher baseline sLOX-1 levels were associated with an increased incidence of recurrent ischaemic events post-operatively. The source of increased sLOX-1 levels and the timing of sampling in acute stroke are areas of controversy. Most studies have hypothesized that sLOX-1 is released from the offending atherosclerotic plaque; however, the contribution of cerebral ischaemia to sLOX-1 levels is unknown. Skarpengland et al. recently examined carotid plaque OLR1 gene expression and plasma sLOX-1 levels in patients referred for carotid endarterectomy [92]. Predictably, circulating sLOX-1 levels and arterial OLR1 expression were increased in patients with carotid plaque compared with healthy controls. Interestingly, sLOX-1 levels were comparably increased in patients with thrombotic ischaemic stroke and embolic stroke secondary to atrial fibrillation, raising the possibility that the ischaemic event may contribute to sLOX-1 release. The embolic stroke patients must have harboured a degree of carotid plaque, however, to warrant a referral for carotid endarterectomy.

The SR member, CXCL16, is a membrane-bound chemokine, which binds oxLDL and promotes proinflammatory responses linked to atherosclerosis development [93]. Serum-soluble CXCL16 (sCXCL16) has been evaluated as a potential biomarker in CAD, inflammatory cardiomyopathy, and carotid artery disease. In 118 patients presenting with an acute ischaemic stroke, serum sCXCL16 levels were significantly higher in patients with vulnerable carotid plaques, a higher degree of luminal stenosis, and increased intimal layer thickness [94]. Other clinical studies have also shown that measuring serum sCXCL16 concentration can improve diagnostic accuracy when combined with cTn and sLOX-1 in ACS, and that may be of benefit in with type 2 diabetes mellitus (T2DM) patients [49,95]. As a prognostic biomarker, a study of 1351 ACS patients found that a single measurement of serum sCXCL16 within 24 h of hospital admission indicated that higher sCXL16 levels increased risk of longer term mortality, suggesting that CXCL16 may be part of sustained proatherogenic inflammatory response in this patient cohort [96]. A recently published cohort study of 5142 ACS patients found that increasing serial CXCL16 levels were associated with composite adverse clinical outcomes and that the CXCL16 level on admission was independently associated with cardiovascular death [97]. A large cohort study of healthy individuals performed in Norway compared baseline CXCL16 levels between those who developed myocardial infarction and those who did not over the trial period of 11 years [98]. Increased baseline CXCL16 levels were associated with increased risk of myocardial infarction, further strengthening the case for CXCL16 as a prognostic biomarker for CAD. CXCL16 has also been proposed as a prognostic biomarker in inflammatory cardiomyopathy and heart failure. In 174 patients with heart failure, myocardial biopsy samples stained for CXCL16 showed significantly enhanced expression in inflammatory cardiomyopathy compared with non-inflammatory cardiomyopathy [99]. The same study found that increased CXCL16 expression was an independent predictor of death in both inflammatory and non-inflammatory cardiomyopathy patients.

The class J members include the receptor for advanced glycation end-products (RAGEs), which can undergo proteolytic cleavage to release a soluble fragment (sRAGE) present in extracellular fluids, including blood. sRAGE has been proposed as a novel diagnostic and prognostic biomarker in CVD; however, conflicting data has led to controversy. Multiple studies have found that increased serum sRAGE levels are predictive of serious clinical events in CAD and cardiovascular conditions [100,101,102,103]. The relationship is not entirely straightforward: a computed tomography angiography study of sRAGE levels in 127 consecutive patients with non-acute CAD found that higher sRAGE levels were inversely proportional to overall plaque burden [104]. Another study of 328 non-diabetic male patients found the same inverse relationship [105]. As a biomarker for the diagnosis of ACS, similar controversy exists. While numerous studies have found significantly higher levels of sRAGE in ACS compared with controls, and a positive correlation between sRAGE and troponin levels in ACS patients, conflicting studies report no significant differences [106]. The most recent study of sRAGE in ACS by Larsen et al. assessed the utility of sRAGE as a prognostic biomarker post-ACS and found that while baseline sRAGE was associated with greater risk of major adverse cardiac events post-ACS, sRAGE levels recorded at 6 weeks post-ACS were inversely related to the rate of recurrent ischaemic events [107]. The impact of diabetes mellitus on the diagnostic utility of sRAGE in ACS has been demonstrated by multiple studies to be non-significant [106].

## 3. SRs as Therapeutic Targets in CVD

### 3.1. Current Therapy

Owing to a central role for SR functionality in atherosclerotic plaque development and progression, targeting SRs is of great interest in CVD therapy. Multiple therapeutic avenues have been explored, including repurposing clinically approved drugs, herbal medicines, and emerging clinical modalities, such as gene therapy and nanoparticle administration. One well-established therapeutic regime worldwide is the long-term use of small-molecule inhibitors of hydroxymethylglutaryl-coenzyme A (HMG Co-A) reductase, commonly known as statins. These drugs are cholesterol-lowering agents that are well accepted in reducing the atherosclerosis burden and risk of CVD [108]. Statins not only reduce total serum cholesterol but also exhibit pleiotropic effects on the vascular wall, including improved endothelial function, a reduction in vascular inflammation, and enhanced plaque stability [109]. The effect of statin therapy on SR function is not yet understood. An in vitro study on a THP-1 human monocyte cell line found that atorvastatin caused dose-dependent attenuation of an oxLDL-stimulated increase in SR-A and monocyte chemoattractant protein-1 (MCP-1) levels [110]. Atorvastatin also reduced lipid-laden foam cell development, indicating that cholesterol levels and plaque development are functionally linked. Another compound, pitavastatin, caused a significant increase in SR-B1 in murine and human macrophages: this SR has been implicated as an anti-atherogenic factor [111,112]. Statins also promote endothelial-derived nitric oxide (NO) levels by activation of SR-B1 and downstream signalling events [112]. Various statins have been found to downregulate CD36 expression in VSMCs in vitro and in circulating monocytes in human subjects [113]. A recent *APO-E*-null mouse model of atherosclerosis found that simvastatin administration reduced CD36 expression as well as atherosclerosis and inflammatory signalling compared with controls [114]. Statins have been shown to downregulate LOX-1 levels and reduce serum sLOX-1 levels, thus reducing the cellular capacity to sense oxLDL, and activate proatherogenic signalling to downstream pathways [115,116]. Reduced LOX-1 levels caused by statin therapy also has additional anti-atherogenic properties, such as increased NO levels, and downregulation of adhesion molecules [117,118]. The importance of the link between LOX-1 and statins in arterial disease is further highlighted in a cohort study of 751 patients with hypercholesterolaemia receiving statin therapy: *OLR1 (LOX-1)* polymorphisms are linked to circulating LDL levels and risk of CVD [119].

Statin administration may also affect proatherogenic RAGE levels. Simvastatin treatment of diabetic patients causes the downregulation of RAGE levels in atherosclerotic plaques [120]. Atorvastatin treatment increases sRAGE levels in vitro. Here, sRAGE could act as a decoy to inhibit proatherogenic effects of advanced glycation end-product (AGE) species [121]. One issue is the structurally different statins have similar effects on SR activity and function; this has not been resolved at present. There are notable differences between the therapeutic effects of atorvastatin, which is less noticeable when pravastatin is used.

Monoclonal antibody proprotein convertase subtilisin/kexin type 9 (PCSK9) inhibitors are relatively novel LDL-lowering agents, which have recently been approved for clinical use. PCSK9 is a protease required for the degradation of the LDLR [122]. PCSK9 inhibitors work by inhibiting PCSK9–LDLR interaction, leading to decreased LDLR degradation and ultimately a reduction in serum LDL cholesterol. Studies by Ding et al. investigating the possibility of interactions between PCSK9 and LOX-1 suggest that cross-talk between PCSK9 and LOX-1 exists in the form of positive feedback [123]. The authors demonstrate LOX-1 ablation in cellular and transgenic mouse experiments results in reduced PCSK9 expression and vice versa with PCSK9 ablation. The activation of the PCSK9–LOX-1 axis has also been shown to be mediated by altered ROS generation at sites of shear stress [124]. The effects of monoclonal PCSK9 inhibitors on LOX-1 expression in humans is not known; however, it is conceivable that LOX-1 downregulation resulting from PCSK9 inhibition could be providing additional anti-atherogenic benefit.

Peroxisome proliferator-activated receptor-γ (PPAR-γ) exhibits anti-inflammatory and antidiabetic properties. Pioglitazone is a PPAR-γ agonist, which is commonly prescribed as a glucose-lowering agent for T2DM patients. Analysis of human and animal models treated with pioglitazone has effects on SR levels and function in the context of diabetes and metabolic syndrome. In a study of 30 patients with polycystic ovarian syndrome (PCOS) combined with either increased fasting glucose or obesity, serum sCD36 levels were significantly raised compared to healthy controls (*n* = 14) [125]. Furthermore, pioglitazone treatment significantly reduced circulating sCD36 and CRP levels whilst improving insulin sensitivity. This strengthens an association between sCD36 and insulin resistance in PCOS. In this cohort, pioglitazone treatment may have an added benefit of reducing risk of CVD. A different study on diabetic *APOE*-null mice subjected to pioglitazone treatment observed attenuation of atherosclerosis via RAGE-linked signalling events [126]. These studies showed that pioglitazone administration reduced atherosclerotic plaque burden in coronary arteries and inhibited RAGE expression in arterial and vascular smooth muscle cells.

Other clinically approved drugs that modulate LOX-1 expression and/or function include ursolic acid, histamine, spironolactone, and angiotensin-converting enzyme (ACE) inhibitors. Ursolic acid inhibits LOX-1 expression in vitro and significantly reduces both LOX-1 expression and atherosclerotic plaque burden in arteries of *APOE*-null mice [127]. Similar outcomes were observed using the same models treated with histamine [128]. Antihypertensive medications, such as spironolactone (potassium-sparing diuretic) and losartan (angiotensin II receptor inhibitor), have been shown to downregulate LOX-1 levels and functionality, thus showing wider benefits for targeting atherosclerosis [129,130]. Nifedipine, a calcium channel blocker used commonly in hypertension, has been found to reduce CD36 expression and lipid internalization in macrophages in vitro [113].

### 3.2. Novel Therapeutics

Deletion of a functional *SR-A* locus in transgenic mouse models causes a significant reduction in atherosclerosis plaque burden in the aorta, stimulating many groups to search for novel therapeutics or inhibitors that target SR-A function or activity [131]. Recent work investigating the role of SR-A activation in neutrophils and NET formation has found that NET release may be triggered by SR-A activation by reactive oxygen species (ROS) [132]. The role of NETs in plaque erosion and thrombo-occlusive complications of CVD is becoming more prominent and the role of SR-A targeting in this area is a potential area for future studies. Certain amphiphilic macromolecules (AMs) that exhibit selective binding to SR-A have shown promise in attenuating atherosclerosis [133,134]. SR-A-specific AMs not only reduce oxLDL binding and uptake in human embryonic kidney cells but also inhibit proinflammatory signalling in activated THP-1 monocytes [134]. Using rat models, this study also showed a reduction in intimal cholesterol accumulation and macrophage retention under proatherogenic conditions [134]. Higher serum levels of the anti-inflammatory cytokine interleukin-10 (IL-10) were associated with superior outcomes in ACS patients, but this was not fully understood at that time [135,136]. Since these studies, it has been found that IL-10 addition reduces oxLDL uptake and downregulates SR-A levels in activated THP-1 monocytes [137]. Such work provides further support for IL-10 as a therapeutic strategy aimed at SR-A for CVD, particularly in ACS.

Macrophage SR-A expression in macrophages is downregulated by treatment with intermedin, a novel member of the calcitonin gene-related peptide family: intermedin attenuates atherosclerosis plaque burden in *APOE*-null mice [138]. Intermedin is implicated in vascular homeostasis by reducing calcium deposition in atherosclerotic plaques [139]. Intermedin treatment caused increased expression of the phosphatase and tensin homolog (PTEN) whilst SR-A levels were reduced, further supporting intermedin use in CVD therapy [139]. SR-A is also a potential target in diabetic nephropathy, although no therapeutics are forthcoming for such therapy. *SR-A*-null mice subjected to streptozotocin-induced diabetes mellitus exhibit significant changes in markers of nephropathy, such as albuminuria, glomerular hypertrophy, mesangial matrix expansion, and parenchymal macrophage infiltration with increased levels of transforming growth factor-β [140].

Using RNA interference (RNAi) to downregulate SR-A and CD36 levels is effective in reducing oxLDL uptake in vitro and atherosclerotic load in vivo when either receptor is targeted in isolation; however, when both receptors are simultaneously targeted, there is no change compared to controls [141]. One likelihood is that downregulation of either SR-A or CD36 alone causes a reciprocal upregulation of the other; SR crosstalk could explain why inhibition of both receptors is less effective than targeting a single receptor. Further studies also indicate conflicting conclusions in targeting both SR-A and CD36 together. Monoclonal antibody-mediated targeting of either SR-A or CD36 separately reduces foam cell formation in vitro [142], but simultaneously targeting both receptors in this context was not studied. Double knockout of *SR-A* and *CD36* in *APO-E*-null mice did not reduce aortic root atherosclerosis compared with *APOE*-null mice; however, the authors did not study *SR-A*- or *CD36*-null mice in isolation [143]. Single and double knockout mouse lines have been studied elsewhere, with findings that *CD36* ablation significantly reduced aortic atherosclerotic plaque burden; however, *SR-A*-null mice were less effective in isolation, and *SR-A/CD36* double-null animals showed comparable efficacy to *CD36*-null mice [144]. A recent study of insulin sensitivity in a mouse model of obesity found that *SR-A* knockout exacerbated insulin resistance compared with wildtype mice [145]. Interestingly, in a similar study, *CD36* knockout provided protective effects against insulin resistance [146]. These studies highlight the importance of studying the off-target effects of SR targeting as each class of receptor is likely to exhibit unique and potentially opposing tissue-specific functions.

CD36 is a multi-ligand multifunctional scavenger receptor, which plays a complex role in CVD and cerebrovascular disease states [147]. CD36 exhibits both proatherogenic and anti-angiogenic properties in vitro and in vivo [148,149] and there is an emerging understanding that there may be an “optimal protective window” of CD36 expression [150]. *CD36*-null mice display a significant increase in angiogenesis and re-vascularization after acute stroke [151]. Treatment with a cell-permeable antioxidant peptide (SS31) reduces cerebral oxidative stress and infarct size in a mice subjected to transient middle cerebral artery occlusion, but this effect was absent in the *CD36*-null group [152], indicating that the therapeutic effects of SS31 is occurring via a CD36-regulated pathway. Nanoparticles, such as AMs and nanovesicles, have been identified, which bind to CD36 and inhibit oxLDL uptake in vitro [68,153]. CD36-targeted liposome-like nanovesicles show impressive binding affinities to CD36 in vitro and in vivo in atherosclerotic *LDLR*-null mice. CD36-targeted nanovesicles thus have potential for reducing atherosclerosis. Synthetic protein ligands that bind CD36, such as EP80317, have been shown to reduce oxLDL uptake by macrophages and atherosclerotic plaque burden in *APOE*-null mice [154]. Furthermore, EP80317 administration promotes reverse cholesterol transport in macrophages; gut receptors, such as LXRα and NPC1L1, further facilitate cholesterol removal through faecal excretion [155]. The administration of ectopic adipokines to ameliorate atherosclerosis by CD36 modulation has been recently investigated by Wang et al. [156]. The authors found that administration of C1q/tumour necrosis factor-related protein 13 (CTRP13) reduced CD36 expression in vitro and significantly reduced aortic atherosclerosis in vivo, thus defining a potential novel therapeutic in atherosclerosis by SR modulation.

LOX-1 targeting in atherosclerosis attracts much interest, due to its proatherogenic properties [157]. In vitro studies show that blocking LOX-1 reduces the proapoptotic effects of oxLDL; transgenic animal studies indicate that *OLR1/LDLR* double-null mice exhibit reduced atherosclerotic plaque burden in coronary arteries [158,159]. Administration of anti-LOX-1 antibody in animal models causes a significant reduction in arterial pathologies characteristic of neointimal hyperplasia and MI [160,161]. Furthermore, anti-LOX-1 antibody administration in a hypertensive rat model causes reduction of lipid deposition in mesenteric arteries; such treatment also attenuates renal vascular fibrosis in an obese diabetic hypertensive rat model [162,163].

Studies on microRNAs (miRNAs) linked to atherosclerosis and CVD is a relatively new but increasing area of interest. These miRNAs are usually tissue-specific relatively small (∼22 bases), non-protein coding RNAs, which bind one or more protein-coding mRNAs to regulate protein expression: miRNAs are implicated in many pathological processes, including atherosclerosis [164]. Dai and colleagues used bioinformatics to identify miRNA-98 as a LOX-1-specific agent and showed that miRNA-98 administration reduced lipid accumulation in vitro; this treatment also reduced LOX-1 expression and aortic plaque burden in *APOE*-null mice [165]. A similar study showed that miRNA let-7g-mediated targeting of LOX-1 attenuated the development of atherosclerosis [166]. As many miRNAs bind multiple mRNA species, off-target effects of miRNA therapy is a serious concern; however, LOX-1-specific miRNA therapy did not raise toxicity issues [165]. Small interfering RNAs (siRNAs), which target the LOX-1 mRNA and knockdown protein expression, can also inhibit LOX-1-regulated pathways in vitro [167]. Although initial studies are promising [167], further evaluation in animal models is needed to better evaluate such treatments to attenuate atherosclerosis and plaque burden. Similar to the use of miRNAs, antibodies, and synthetic proteins to target LOX-1, careful evaluation of off-target effects and absorption, distribution, metabolism, excretion, and toxicity (ADMET) parameters is needed before effective therapies can be implemented [168].

The three-dimensional structure of the LOX-1 extracellular domain has been determined using a combination of crystallography and NMR [169]. This 3-D model for LOX-1 was used to screen chemical libraries to identify new molecules that block LOX-1 function; two lead compounds could reduce oxLDL uptake and monocyte adhesion to endothelial cells [170]. Chemical compounds with cell- and tissue-penetrating properties have substantial advantages over antibody or synthetic protein-based therapies; however, such compounds require careful evaluation of ADMET parameters in whole animal models. Single-chain variable fragment (scFv) antibodies targeting LOX-1 have been recently reported [171]. These scFvs exhibit specific binding affinity, enhanced bio-distribution, and low immunogenicity compared with humanized monoclonal antibodies, whilst being substantially cheaper to produce using bacterial expression systems. LOX-1-specific scFvs are in the early stages of development and ongoing studies are aiming to improve the binding affinity, thermostability, and serum half-life, and using multimerization techniques [172,173].

The relationship between CXCL16 and atherogenesis is less defined compared with SR-A, CD36, and LOX-1. A sCXCL16 isoform is a potential biomarker for CAD, and membrane-bound CXCL16 is highly expressed in carotid and coronary artery atheromas [174]. Hofnagel and co-workers found that CXCL16 is expressed primarily in the endothelium at sites predisposed to atherosclerosis in the rabbit aorta; furthermore, anti-CXCL16 antibody treatment of primary human endothelial cells caused a significant reduction in endothelial-monocyte adhesion [175]. However, there is still a need to demonstrate that anti-CXCL16 monoclonal antibody therapy has efficacy in an animal model of atherosclerosis. Interestingly, *LDLR/CXCL16* double-null transgenic mice displayed an increased atherosclerotic plaque burden compared to *LDLR*-null mice [93]; this work suggests that the *CXCL16* locus is anti-atherogenic, contradicting previous studies.

AGEs have been suggested to be proatherogenic, especially in the context of atherosclerosis in diabetes mellitus patients. Inhibition in biochemical pathways that cause the production of AGEs using agents, such as aminoguanidine-HCl, which inhibits chemical crosslinking, can produce beneficial cardiovascular outcomes in vivo [176]. The binding and cellular uptake of oxLDL by RAGE is promoted by AGEs; this proatherogenic effect can be blocked by anti-RAGE treatment [177]. RNAi to target RAGE expression reduced endothelial-monocyte adhesion, and improved CVD-related outcomes [178]. Diabetic transgenic mice carrying the *APOE/RAGE* double-null genotype displayed reduced leucocyte adhesion, expression of proinflammatory mediators, and a statistically non-significant decrease in reduction in aortic atherosclerotic plaque burden [179]. Interestingly, administration of recombinant sRAGE in diabetic *APOE*-null mice suppresses atherosclerosis whilst having little or no effect on plasma lipids or serum glucose levels [180]. One hypothesis is that sRAGE acts as a circulating soluble “AGE sponge”, thus downregulating membrane-bound RAGE expression in vascular tissues, further reducing proatherogenic signalling and cellular responses [180].

Novel drug delivery systems, such as nanocarriers and nanovesicles, have been employed with some success in SR targeting in atherosclerosis [68,153]. The ideal delivery system is one that exhibits immunological inertness and non-cytotoxicity, high tissue permeability and specificity, excellent biodistribution, and adequate half-life in the circulation. A recently published study evaluating the use of M2 macrophage-derived exosomes as a novel drug delivery system in atherosclerosis describes their ability to target areas of atherosclerosis by inflammation-tropism via the expression of surface-bonded chemokine receptors [181]. Atherosclerosis-targeting drug delivery systems may have an important future role in the development of SR-targeted therapies.

## 4. Conclusions

SRs are a multi-ligand receptor super-group defined by the ability to recognize modified lipid particles; each class has a defined consensus sequence, which is usually unrelated to other classes. One common feature of this diverse group of proteins is the ability to bind potentially harmful substances to mediate clearance from circulation. Many SRs are linked to atherosclerosis, which is the main pathway involved in the development of CVD. The current view is that the main SRs involved in CVD are SR-A, CD36, LOX-1, CXCL16, and RAGE. Monitoring serum levels of soluble SRs, alongside cardiac troponins, in better diagnosis of MI and ACS is a potential prognostic tool. Current cholesterol-lowering therapies, e.g., statin treatment, have been also found to modulate SR expression and downstream signalling, suggesting wider implications of SR function linked to cholesterol metabolism. So far, numerous approaches for targeting SRs, including gene therapy, monoclonal antibodies, and nanoparticles, have shown differing levels of success in reducing CVD. A major issue is that SR studies have so far not progressed beyond animal models; only SR biomarker assessments in human patients have been carried out. We concur that the future application of SR-linked tools and therapies will play a central role in the diagnosis, management, and treatment of arterial pathologies, such as CVD.

## 5. Future Perspectives

SRs are attractive molecular targets in the development of targeted therapy for atherosclerosis. While the results from in vitro and in vivo models are promising, SR receptor modulation in humans has not been investigated. Given that SRs expression is widespread in multiple organ systems, the concept of systemic SR modulation should be considered with caution as there is a significant potential for off-target effects and reciprocal upregulation of SRs from the other classes. Local delivery of SR modulators utilizing novel delivery systems, such as exosomes and other nanocarrier technologies, may play an important role in translating SR-targeting agents to a clinical setting.

## Figures and Tables

**Table 1:** SR expression and function.

SR Class	Nomenclature/Isoforms	Expression	Function
SR-A	Macrophage SR	Macrophages and subtypes e.g., Kupffer cells, medullary thymic macrophages Vascular smooth muscle cells Endothelial cells Microglia [26,27,28]	Internalization of oxLDL in atherosclerosis. Bacterial cell recognition in innate immunity. Involved in beta-amyloid clearance in the brain. [20,29,30,31,32]
SR-B	SR-B1 SR-B2 (CD36) SR-B3 (LIMP2)	Macrophages Endothelial cells Adipocytes Renal tubular cells Podocytes [33,34]	Internalization of oxLDL in atherosclerosis. Bacterial cell adhesion, internalization and lysosomal sequestration. May participate in systemic inflammation associated with sepsis. [23,35,36]
SR-C	Not discussed (plant receptor)		
SR-D	CD68	Macrophages Monocytes Microglia Osteoclasts Myeloid dendritic cells [37]	May play a role in internalization of oxLDL. [38,39,40]
SR-E	SR-E1 (LOX-1) SR-E1.1 (LOXIN) SR-E2 (Dectin-1)	Macrophages Neutrophils Endothelial cells Smooth muscle cells Platelets [41]	Internalization of oxLDL in atherosclerosis. Bacterial and fungal cell recognition in innate immunity. LOXIN (alternatively spliced form of SR-E1) demonstrates no known scavenger receptor activity but has been shown to exert a dominant negative effect on LOX-1 function. [41,42]
SR-F	SR-F1 (SREC-1,SCARF-1) SR-F2 (SREC-2, SCARF-2) MEGF10	Neuronal cells Sinusoidal Endothelial cells [19,43]	SR-F1 binds and internalizes oxLDL, whilst formation of heterodimer with SR-F2 suppresses oxLDL binding activity. MEGF10 is involved in beta-amyloid clearance in the brain. [21,43,44]
SR-G	SR-G1 (CXCL16, SR-PSOX)	Macrophages Smooth muscle cells [45,46]	Adhesion of cells expressing the CXCR6 receptor such as natural killer T cells and polarized T helper cells. Internalization of oxLDL in atherosclerosis. Bacterial cell recognition in innate immunity. [47,48,49]
SR-H	SR-H1 (FEEL-1, Stabilin-1, Clever-1) SR-H2 (FEEL-2, Stabilin-2)	Macrophages Splenic, hepatic and lymphatic endothelial cells Monocytes [50,51]	Internalization of oxLDL in atherosclerosis. Bacterial cell recognition in innate immunity. Binding of advanced glycation end-products (AGEs). Potential role in the adhesion of metastatic tumour cells to lymphatic endothelial cells. [50,52,53]
SR-I	SR-I1 (CD163, haemoglobin SR) SR-I2 (CD163B)	Circulating and tissue specific macrophages and monocytes Leukemic blasts [54,55]	Mediates haptoglobin-haemoglobin complex endocytosis during intravascular haemolysis. [55]
SR-J	RAGE	Endothelial cells Hepatocytes Smooth muscle cells Monocytes [56]	Binds AGEs. Amplification of immune and inflammatory responses, cell mobility, arterial injury, and atherogenesis via sustained post-receptor signalling. [57]

**Table 2:** SRs as biomarkers in CVD.

Disease State	SR Biomarker	Human Studies
CAD/ACS	SR-A	SR-A index (monocyte SR-A expression in peripheral blood film) associated with advanced plaque morphology [63,64,65]. SR-A-targeted probes used for plaque imaging [67].
	SR-B	Monocyte CD36 expression associated with increased atheroma burden in CAD patients [66]. CD36-targeted probes used for plaque imaging [68]. Baseline sCD36 not associated with CAD development or plaque burden [70,71].
	SR-E	Raised serum levels of sLOX-1 used as a diagnostic biomarker in ACS [75,76,77]. sLOX-1 levels combined with troponin levels provide more diagnostic accuracy compared with either in isolation [76,77]. Raised sLOX-1 levels correlate with incidence of major adverse cardiovascular events in stable CAD [79].
	SR-G	sCXCL16 levels improve diagnostic accuracy in ACS when combined with troponin/LOX-1 [49,95]. sCXCL16 levels are associated with increased adverse events and mortality in ACS [96,97]. Baseline sCXCL16 levels associated with increased risk of ACS in non-CAD patients [98].
	SR-J	Due to conflicting reports, the potential use of sRAGE as a biomarker for ACS remains controversial.
Cardiomyopathy	SR-E	sLOX-1 levels negatively correlate with left ventricular ejection fraction [84].
	SR-G	Increased sCXCL16 levels independently predict mortality in inflammatory and non-inflammatory cardiomyopathy [99]. Enhanced CXCL16 expression in cardiac myocytes in inflammatory cardiomyopathy [99].
Stroke	SR-E	sLOX-1 levels increased in ischaemic and haemorrhagic stroke [88,89]. Increased baseline sLOX-1 levels associated with increased incidence of ischaemic stroke and secondary ischaemic events post-carotid intervention [91].
	SR-G	Increased sCXCL16 associated with more advanced carotid lesions in patients presenting with ischaemic stroke [94].
